# Single Amino Acid Substitution in Homogentisate Dioxygenase Affects Melanin Production in *Bacillus thuringiensis*

**DOI:** 10.3389/fmicb.2018.02242

**Published:** 2018-10-11

**Authors:** Wenjun Yang, Lifang Ruan, Jiangming Tao, Donghai Peng, Jinshui Zheng, Ming Sun

**Affiliations:** ^1^State Key Laboratory of Agricultural Microbiology, College of Life Science and Technology, Huazhong Agricultural University, Wuhan, China; ^2^College of Informatics, Huazhong Agricultural University, Wuhan, China

**Keywords:** *Bacillus thuringiensis*, tyrosine catabolism, homogentisate dioxygenase, site-directed mutagenesis, melanin

## Abstract

*Bacillus thuringiensis* formulation losing its activity under field conditions due to UV radiation and photoprotection of *B. thuringiensis* based on melanin has attracted the attention of researchers for many years. Here, a single amino acid substitution (G272E) in homogentisate 1,2-dioxygenase was found to be responsible for pigment overproduction in *B. thuringiensis* BMB181, a derivative of BMB171. Disrupting the gene encoding homogentisate dioxygenase in BMB171 induced the accumulation of the homogentisic acid and provoked an increased pigment formation. To gain insights into homogentisate 1,2-dioxygenase in *B. thuringiensis*, we constructed a total of 14 mutations with a single amino acid substitution, and six of the mutant proteins were found to affect the melanin production when substituted by alanine. This study provides a new way to construct pigment-overproducing strains by impairing the homogentisate dioxygenase with a single mutation in *B. thuringiensis*, and the findings will facilitate a better understanding of this enzyme.

## Introduction

*Bacillus thuringiensis*, a gram-positive spore-forming soil bacterium, has been widely used in biological pest control due to the formation of parasporal crystal proteins that are toxic to the larvae of various insect pests (Sudakin, 2003). However, the insecticidal activity of the crystal proteins would be reduced or destroyed under field conditions because of UV damage in sunlight (Pusztai et al., 1991). To solve this problem, researchers have proposed a series of strategies to protect insecticidal crystal proteins from UV damage (Sanchis et al., 1999; Manasherob et al., 2002; Jallouli et al., 2014), and one of them is the use of melanin, a photoprotective agent, to reduce the damaging effect of UV radiation on *B. thuringiensis* toxins (Liu et al., 1993; Ruan et al., 2002; Zhang et al., 2008; Sansinenea and Ortiz, 2015).

Melanins are polymers of phenolic and/or indolic compounds and classified into three main categories: eumelanins, pheomelanins, and allomelanins (Plonka and Grabacka, 2006). These black pigments are widely distributed in nature and can be found in species of all biological kingdoms, including humans, fungi, and bacteria (Plonka and Grabacka, 2006). Melanins provide free-living species a survival advantage in the environment by protecting against different exogenous stresses, such as UV-irradiation, reactive oxygen species (ROS), metals, and defensins (Nosanchuk and Casadevall, 2003, 2006; Heinekamp et al., 2012). Both eumelanins and pheomelanins are produced from the oxidation of tyrosine or phenylalanine to o-dihydroxyphenylalanine (DOPA) and dopaquinone via tyrosinases or laccases. Allomelanins include a heterogeneous group of polymers formed through the oxidation and polymerization of the intermediates such as dihydroxynaphthalene, homogentisic acid (HGA), γ-glutaminyl-4-hydroxybenzene, catechols, and 4-hydroxyphenylacetic acid (Plonka and Grabacka, 2006).

Homogentisic acid is derived from the tyrosine or phenylalanine catabolism pathway (one branch of tyrosine metabolism) and further oxidized to acetoacetic acid and fumaric acid (Turick et al., 2010). Pyomelanin is formed from the autoxidation and self-polymerization of HGA with the deactivation of homogentisate dioxygenase (HmgA) or the disruption of the gene encoding HGA-oxidase (Rodriguez-Rojas et al., 2009; Schmaler-Ripcke et al., 2009; Valeru et al., 2009). A deficiency of this enzyme in humans causes the metabolic disease alkaptonuria (AKU), leading to the excretion of HGA in the urine in a large amount and its deposition in different tissues (Millucci et al., 2012). The synthesis of pyomelanin has been investigated in a broad range of bacteria, such as *Aeromonas media, Burkholderia cepacia*, *Bacillus anthracis*, *Legionella pneumophila*, *Pseudoalteromonas*, *Pseudomonas aeruginosa*, *Pseudomonas putida*, *Ralstonia solanacearum*, *Shewanella colwelliana*, and so on (Arias-Barrau et al., 2004; Valeru et al., 2009; Turick et al., 2010; Gonyar et al., 2015; Han et al., 2015; Wang et al., 2015; Ahmad et al., 2017; Zeng et al., 2017).

A number of studies have been performed to improve the photoprotection of the *B. thuringiensis* crystal proteins by producing melanin or increasing the melanin yield in *B. thuringiensis* cells. Some studies focused on screening *B. thuringiensis* mutants that can produce melanin in different conditions, and others attempted to construct recombinant *B. thuringiensis* strains with melanin production by genetic engineering (Hoti and Balaraman, 1993; Ruan et al., 2002; Saxena et al., 2002; Zhang et al., 2008; Sansinenea and Ortiz, 2015). It has been found that melanin could be produced by most *B. thuringiensis* strains in the presence of L-tyrosine at an elevated temperature (42°C), but the insecticidal Cry proteins could not be synthesized at this temperature (Ruan et al., 2004). In our previous work, a mutant, *B. thuringiensis* strain BMB181, was identified to be able to produce the brownish black pigment as an alternative melanin without tyrosine supplementation in the growth medium, and this strain could achieve a high melanin yield in different media without additional L-tyrosine (Liu et al., 2013). However, the mechanism for melanin production by BMB181 remains unclear. Here, the pigment produced by the strain BMB181 was found to be derived from homogentisate acid. The inactivation of HmgA by a G272E amino acid substitution resulted in pigmentation in the strain BMB181. Six single-point mutations in HmgA resulted in observable changes of melanin production in *B. thuringiensis*. This study offers valuable information about HmgA and provides a new way of constructing the pigment production strain of *B. thuringiensis*.

## Materials and Methods

### Bacterial Strains, Plasmids, and Growth Conditions

The bacterial strains and plasmids used in this study are shown in **Table 1**. The *B. thuringiensis* strain BMB171 and its derivative BMB181 have been reported in previous studies (He et al., 2010; Liu et al., 2013). Bacteria were grown in Luria-Bertani (LB) medium at 37°C (*E. coli*) or 28°C (*B. thuringiensis*) and 200 rpm. The antibiotics, including kanamycin (50 μg/mL), erythromycin (25 μg/mL), and ampicillin (100 μg/mL), were added when necessary.

**Table 1:** Bacterial strains and plasmids used in this study.

Strains or plasmids	Characteristics^a^	Origin or reference
***Escherichia coli***		
DH5α	*supE*44 Δ*lacU*169 (φ80*lacZ*ΔM15) *hsdR*17 *recA*1 *endA*1 *gyrA*96 *thi*-1 *relA*1	Stored in this lab
***Bacillus thuringiensis***		
BMB171	A crystalliferous mutant of *B. thuringiensis*	He et al., 2010
BMB181	A mutant strain of BMB171, produce the brownish black pigment without tyrosine supplementation in the growth medium	Liu et al., 2013
BMB171Δ*hmgA*	BMB171 derivative, with a kanamycin insertion in *hmgA* gene	This work
BMB3141	derivative of BMB181, containing pBMB3141	This work
BMB3142	derivative of BMB181, containing pBMBL	This work
BMB3143	derivative of BMB171Δ*hmgA*, containing pHT304	This work
BMB3144	derivative of BMB171Δ*hmgA*, containing pBMB3144	This work
BMB3145	derivative of BMB171Δ*hmgA*, containing pBMB3145	This work
BMB3146a	derivative of BMB181, containing pBMB3144	This work
BMB3146b	derivative of BMB181, containing pBMB3145	This work
**Plasmids**		
pHT304	*E. coli* and *B. thuringiensis* shuttle vector; Amp^r^, Erm^r^	Arantes and Lereclus, 1991
pBMBL	Derivative of pHT304, containing a sporulation-dependent promoters BtI-BtII and the *cry1Ac* transcription terminator, Amp^r^, Erm^r^ ≈7.6 kb	Stored in this lab, unpublished
pDG780	*E. coli* vector, Amp^r^, Kan^r^	Guerout-Fleury et al., 1995
pHT304-ts	Derivative of pHT304, containing a temperature-sensitive replicon in *B. thuringiensis*, ≈6.8 kb, Amp^r^, Erm^r^	Liu et al., 2010
pBMB3141	Derivative of pBMBL, containing the *hmgA* gene (1,173 bp) from BMB171, Amp^r^, Erm^r^	This work
pBMB3143	Derivative of pHT304-ts, a plasmid containing the *hmgA* gene fragments flanking the kanamycin resistance cassette Amp^r^, Erm^r^, Kan^r^	This work
pBMB3144	Derivative of pHT304, carrying the *hmgA* gene operon from BMB171	This work
pBMB3145	Derivative of pHT304, carrying the *hmgA* gene operon from BMB181	This work
pBMB3146	Derivative of pHT304, containing the transcription promoter and terminator of *hmgA* gene operon, the gene of *hmgA* from BMB171	This work

^a^*Amp^*r*^, ampicillin resistance; Erm^*r*^, erythromycin resistance; Kan^*r*^, kanamycin resistance*.

### DNA Manipulation and Sequence Analysis

In this study, DNA was manipulated using the standard techniques as previously described (Green and Sambrook, 2012). The DNA of *B. thuringiensis* was extracted as previously reported (Andrup et al., 1993). The DNA fragments were amplified with the related primers, and the polymerase chain reaction (PCR) products were confirmed by DNA sequencing. The DNA sequences were analyzed using the DNASTAR software and the protein sequences were compared with those of other proteins using BLAST and CLUSTALX.

### Transformation Techniques

*Escherichia coli* was transformed using CaCl_2_-treated competent cells (Green and Sambrook, 2012), and *B. thuringiensis* was transformed by electroporation with the Bio-Rad Gene Pulser set (Bio-Rad, Hercules, CA, United States) (Peng et al., 2009).

### High Performance Liquid Chromatography (HPLC) Analysis of Culture Filtrates

The HPLC analysis of culture filtrates was performed using an HPLC apparatus equipped with a variable wavelength UV-visible detector (CapLC 2487, Waters) and a C-18 end-capped column (10 μm, 4.6 mm × 150 mm; Elite). Briefly, the bacteria were grown in LB medium at 28°C and 200 rpm, and the culture supernatants were collected by centrifugation and filter sterilization. Next, 20 μL of the sample was injected into the column for HPLC. The mobile phase was 50 mM sodium phosphate buffer (pH 6.5)/methanol (80:20, v/v) at a flow rate of 0.5 mL/min as described by Fernandez-Canon and Penalva (1995). The chromatograms of standard solutions of HGA (from Sigma) were used as a reference to identify the corresponding HPLC peaks. The absorption maximum of HGA is 290 nm.

### Isolation of the *hmgA* Gene

The *hmgA* gene (1,173 bp) encoding HmgA was amplified from *B. thuringiensis* BMB171 and BMB181 genomic DNA using the pair of primers, hmgA1 (5′-CGCGGATCCATGTTTTATCGTCACATGGGAG-3′, with the *Bam*HI recognition sequence underlined) and hmgA2 (5′-CGCGTCGACTTATTTCACAGTATATGAACCT-3′, with the *Sal*I recognition sequence underlined). The *hmgA* genes from BMB171 and BMB181 were designated as 171*hmgA* and 181*hmgA*, respectively.

### Insertional Inactivation of the *hmgA* Gene

To verify the function of the *hmgA* gene, the gene disruption strain was constructed for BMB171 via homologous recombination using a temperature-sensitive shuttle vector pHT304-ts containing the temperature-sensitive replication origin (Liu et al., 2010). Briefly, a 503-bp fragment and a 600-bp fragment, corresponding to the DNA regions upstream and downstream of the open reading frame of the *hmgA* gene in the strain BMB171, were generated by PCR using the primer pairs HmgA-up-1(5′-CGCGGATCCTGGGAGAACTACCTCATAAAC-3′)/HmgA-up-2(5′-CCGGAATTCGCTATTCGCCTCTACAACA-3′) and HmgA-down-1(5′-CCGGTCGACAATTGTTAGAGCATAGTCCG-3′)/HmgA-down-2(5′-CGGGGTACCATGAACCTTGTTCAATCCAG-3′) and digested with *Bam*HI- *Eco*RI and *Acc*I-*Kpn*I, respectively. A kanamycin resistance cassette (1,514 bp) was acquired by digesting plasmid pDG780 (Guerout-Fleury et al., 1995) with *Eco*RI-*Acc*I. These three fragments were cloned into the plasmid pHT304-ts at the *Bam*HI-*Kpn*I site. The resulting plasmid, pBMB3143, was transformed into the strain BMB171 by electroporation.

The mutants were selected as follows. Specifically, the transformants were cultured in LB medium with kanamycin (50 μg/mL) for 8 h, followed by incubation at 42°C for 4 days to eliminate unintegrated temperature-sensitive plasmids. Finally, the mutant strains that were resistant to kanamycin but sensitive to erythromycin colonies were confirmed by PCR using appropriate primers and sequencing.

### Genetic Complementation Analysis

For genetic complementation analysis, complementation plasmids were prepared by using the shuttle vector pHT304 (Arantes and Lereclus, 1991) and its derivative pBMBL (unpublished data) that contained a sporulation-dependent promoter BtI-BtII and the *cry1Ac* transcription terminator. The amplified fragment *171hmgA* was cloned into the vector pBMBL to yield the plasmid pBMB3141. To construct the plasmids pBMB3144 (carrying 171*hmgA* operon) and pBMB3145 (carrying 181*hmgA* operon), the *hmgA* gene operon region (about 3.9 kb) containing the genes encoding 4-hydroxyphenylpyruvate dioxygenase (HppD), HmgA, and fumarylacetoacetate hydrolase (FahA) was amplified, respectively, from the strains BMB171 and BMB181 using special primers HMGAop-S (5′-CGCGGATCCAGATATATAAATACAATCATTC-3′, with the *Bam*HI recognition sequence underlined) and HMGAop-A (5′-CGCGTCGACTCTTTCACTCCTCCAAGTTT-3′, with the*Sal*I recognition sequence underlined) and subsequently cloned into pHT304. Finally, the two recombinant plasmids were transformed into the pigmented *B. thuringiensis* strains by electroporation separately.

### Measurement of the Bacterial Growth Curve and the Pyomelanin Production

The growth curve and the pigment production of the *B. thuringiensis* strains and their derivatives were evaluated according to optical density (OD). To monitor the growth curves of the strains, the bacteria were inoculated to 100 mL of LB medium (the flask volume is 500 mL) and incubated under shaking at 28°C and 200 rpm, followed by the OD measurement of the cultures at 600 nm (OD_600_) at different time intervals. The melanin production of the strains was quantified by testing the absorbance of the centrifuged culture supernatant at 400 nm (OD_400_) at the indicated time points (Ruan et al., 2002; Liu et al., 2013).

### Alanine Scanning Site-Directed Mutagenesis

For performing alanine scanning site-directed mutagenesis, we constructed the plasmid pBMB3146 (a derivative of pHT304, containing the promoter region and the terminator region of the *hmgA* gene operon and the 171*hmgA* gene). Briefly, the promoter region of the *hmgA* gene operon (394 bp) was amplified from the strain BMB171 genomic DNA using the pair of primers, HMGAop-S (5′-CGCGGATCCAGATATATAAATACAATCATTC-3′, with the *Bam*HI recognition sequence underlined) and HMGAop-A2 (5′-CTCCCATGTGACGATAAAACATAATATCTTCATCTCCCTGTAA-3′). Next, the 1,405-bp PCR product, containing the 171*hmgA* gene and the terminator region of the *hmgA* gene operon, was amplified using primers HMGA-S2 (5′-TTACAGGGAGATGAAGATATTATGTTTTATCGTCACATGGGAG-3′) and HMGAop-A (5′-CGCGTCGACTCTTTCACTCCTCCAAGTTT-3′, with the *Sal*I recognition sequence underlined). Finally, the vector pBMB3146 was generated by connecting the two fragments with overlapping PCR (Higuchi et al., 1988) using primers HMGAop-S and HMGAop-A, digesting the overlapping PCR fragment with *Bam*HI and *Sal*I, and inserting it into the shuttle vector pHT304.

A total of fourteen amino acid residues of HmgA were mutated to alanine residues by overlapping PCR using the primers shown in **Table 2**, with vector pBMB3146 as the template. The PCR fragments were cloned separately into the vector pHT304 between the *Bam*HI and *Sal*I restriction sites. The recombinant plasmids were transformed into *E. coli* DH5a and the positive clones were screened by restriction enzyme analysis. Before transformation into the pigmented *B. thuringiensis* strain BMB171Δ*hmgA*, all the resulting mutant plasmids were sequenced to ensure that the proper mutations were maintained.

**Table 2:** Primers used for site-directed mutagenesis.

Primers	Oligonucleotides (5′→3′)^a^	Use
HMGAop-S	CGCGGATCCAGATATATAAATACAATCATTC	
HMGAop-A	CGCGTCGACTCTTTCACTCCTCCAAGTTT	
Hm89-R	GTGATGCAATAAGT**GCA**CGGAATTTCATACT	G89A substitution
Hm89-L	AGTATGAAATTCCG**TGC**ACTTATTGCATCAC	
Hm116-R	ATTATTTCTATCGT**GCA**GGTGATGGCGACGA	N116A substitution
Hm116-L	TCGTCGCCATCACC**TGC**ACGATAGAAATAAT	
Hm119-R	ATCGTAATGGTGAT**GCA**GACGAAATGTTATT	G119A substitution
Hm119-L	AATAACATTTCGTC**TGC**ATCACCATTACGAT	
Hm120-R	GTAATGGTGATGGC**GCA**GAAATGTTATTTGT	D120A substitution
Hm120-L	ACAAATAACATTTC**TGC**GCCATCACCATTAC	
Hm128-R	TATTTGTTCATTAT**GCA**ACAGGGAAAATTGA	G128A substitution
Hm128-L	TCAATTTTCCCTGT**TGC**ATAATGAACAAATA	
Hm136-R	AAATTGAAACGATG**GCA**GGAACGATTCACTA	F136A substitution
Hm136-L	TAGTGAATCGTTCC**TGC**CATCGTTTCAATTT	
Hm219-R	TTGTCGTAATGACA**GCA**TCAAGAGGCTATAT	K219A substitution
Hm219-L	ATATAGCCTCTTGA**TGC**TGTCATTACGACAA	
Hm241-R	TTGTGGGATGGGAT**GCA**TATTTATATCCGTG	G241A substitution
Hm241-L	CACGGATATAAATA**TGC**ATCCCATCCCACAA	
Hm245-R	ATGGCTATTTATAT**GCA**TGGGTATTTAATGT	P245A substitution
Hm245-L	ACATTAAATACCCA**TGC**ATATAAATAGCCAT	
Hm261-R	TTACAGGGCGCATT**GCA**CAGCCACCGCCAGT	H261A substitution
Hm261-L	ACTGGCGGTGGCTG**TGC**AATGCGCCCTGTAA	
Hm300-R	CATATTATCATAGT**GCA**GTTAATAGTGATGA	N300A substitution
Hm300-L	TCATCACTATTAAC**TGC**ACTATGATAATATG	
Hm323-R	AAGGTGTGGAAGAA**GCA**TCTATTACACTTCA	G323A substitution
Hm323-L	TGAAGTGTAATAGA**TGC**TTCTTCCACACCTT	
Hm334-R	CGAGCGGGATTCCC**GCA**GGACCGCATCCGGG	H334A substitution
Hm334-L	CCCGGATGCGGTCC**TGC**GGGAATCCCGCTCG	
Hm336-R	GGATTCCCCATGGA**GCA**CATCCGGGGAAAAC	P336A substitution
Hm336-L	GTTTTCCCCGGATG**TGC**TCCATGGGGAATCC	

^a^*The restriction sites included in the oligonucleotide sequences are underlined. Nucleotide codons encoding the mutated amino acids are highlighted in bold types*.

### Sequence Accession Number

The sequences reported in this paper have been submitted to the GenBank. The accession number for the complete genome of the strain BMB171 is CP001903.1. The accession number for the HmgA (locus tag is BMB171_C0216) is ADH05034.1.

## Results

### Pigment Results From Polymerization of Homogentisate

**FIGURE 1:**
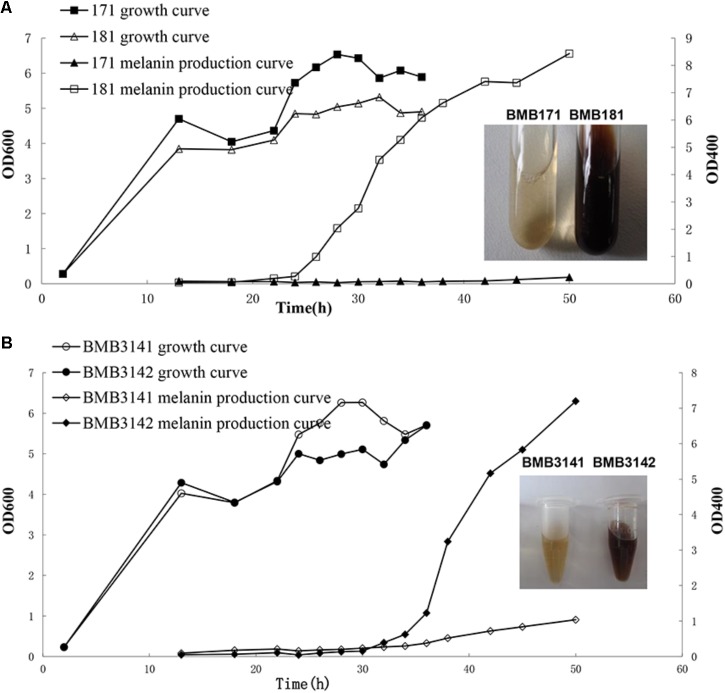
Growth and melanin production curves of *B. thuringiensis* strains in LB medium. **(A)** Growth and melanin production curves of BMB171 and BMB181 in LB medium. **(B)** Growth and melanin production curves of BMB3141 and BMB3142 in LB medium. BMB3141, a derivative of BMB181, contained pBMB3141 that harbors 171*hmgA*; BMB3142, a derivative of BMB181, contained pBMBL, as a negative control.

**FIGURE 2:**
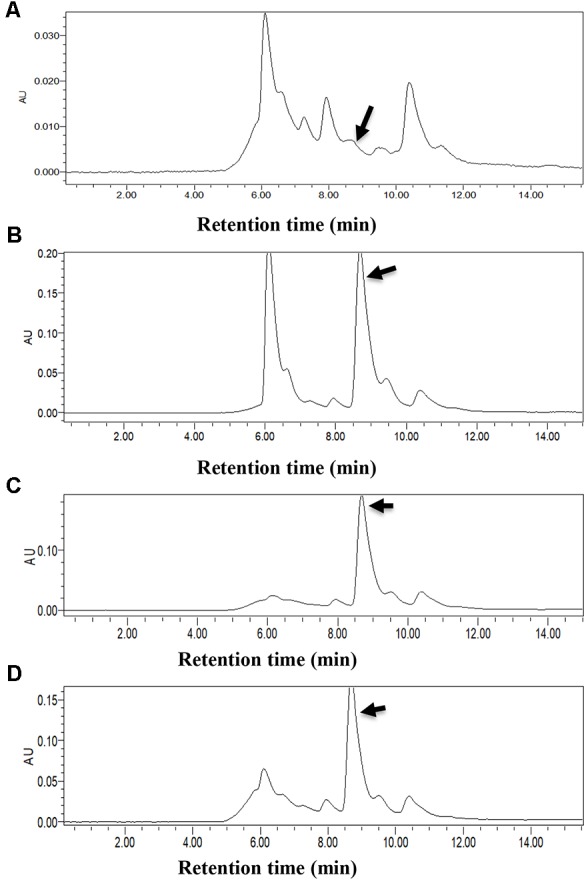
HPLC analysis of culture filtrates of BMB171, BMB181, and BMB171Δ*hmgA*. Homogentisate acid was identified by HPLC and is indicated by black arrows. **(A)** Supernatants of BMB171 (with ascorbic acid), as a negative control. **(B)** Supernatants of BMB171 (with ascorbic acid and Homogentisate), as positive control. **(C)** Supernatants of BMB181 (with ascorbic acid). **(D)** Supernatants of BMB171Δ*hmgA* (with ascorbic acid).

BMB181, a *B. thuringiensis* mutant with high melanin production, was obtained after subculturing the strain BMB171 for several generations at 42°C (Liu et al., 2013). The red pigment produced by the strain BMB181 turned dark brown with the extension of incubation time (**Figure 1A**). Pigments can be formed from the oxidation and polymerization of compounds such as DOPA and HGA (Plonka and Grabacka, 2006). In our early work, we found that the pigment produced by the strain BMB181 has nothing to do with DOPA (data not shown). Therefore, we test whether HGA is the precursor of pigment produced by the strain BMB181. Here, ascorbic acid was added as an antioxidant to prevent HGA from oxidation. No pigment was observed when ascorbic acid (2 mM) was added to the cultures of the strain BMB181 (data not shown). Strains BMB171 and BMB181 were cultured in LB liquid media under shaking at 28°C for 24 h and the culture samples were taken for HPLC analysis after centrifugation and filtration, using the commercially available HGA as the standard. A peak corresponding to HGA (with a retention time of 8.68 min) was identified in the culture supernatants of the strain BMB171 with HGA added to the culture during the logarithmic growth phase (**Figures 2A,B**). The peak with the same chromatography retention time as HGA was identified in the culture supernatants of the strain BMB181 (**Figure 2C**), suggesting that homogentisate could be secreted by the pigmented strain BMB181 and the pigment produced by the strain probably resulted from the accumulation and polymerization of homogentisate.

### Identification of Amino Acid Substitution in HmgA

**FIGURE 3:**
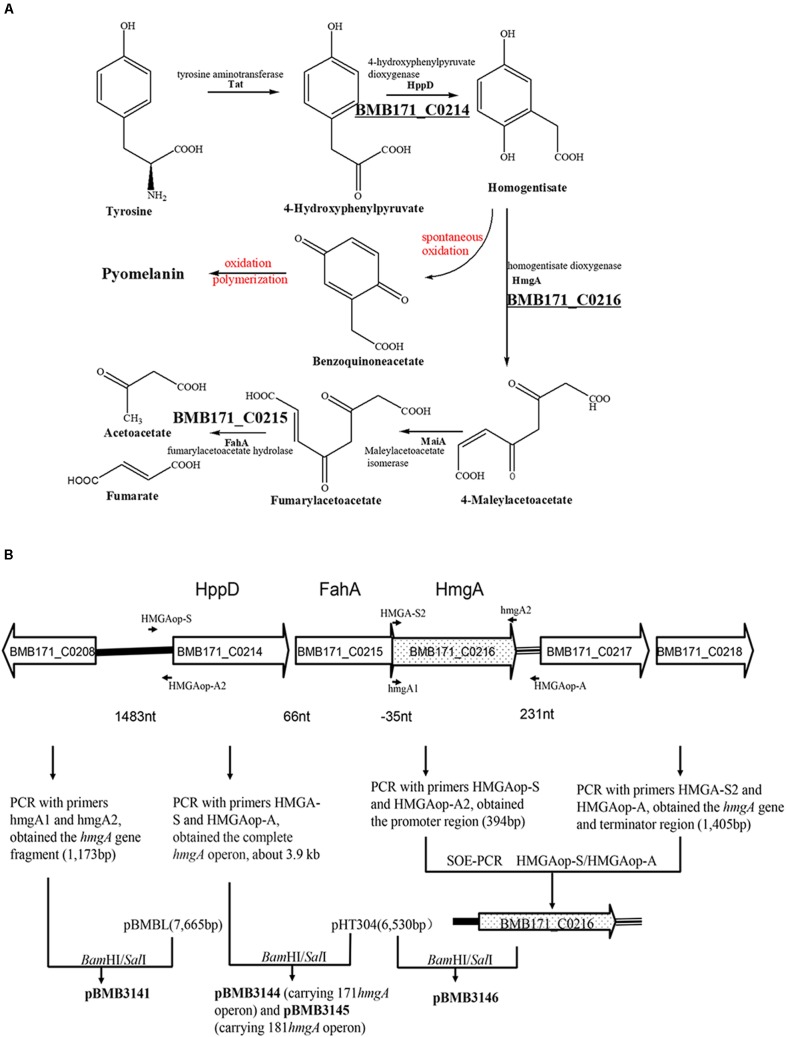
Pathway for the catabolism of homogentisate. **(A)** L-Tyrosine metabolism pathway via homogentisate [modified from reference (Schmaler-Ripcke et al., 2009)]. **(B)** Organization of the genes putatively involved in the catabolism of homogentisate in *B. thuringiensis* BMB171 and scheme for the construction of plasmid vectors. The black arrows indicate the primers used here. Enzymes encoded by the respective genetic loci in *B. thuringiensis* BMB171 are BMB171_C0208, HAD superfamily hydrolase; BMB171_C0214, HppD, 4-hydroxyphenylpyruvate dioxygenase; BMB171_C0215, FahA, fumarylacetoacetate hydrolase; BMB171_C0216, HmgA, homogentisate 1,2-dioxygenase; BMB171_C0217, amino acid permease; BMB171_C0218, MFS transporter. The vector pBMB3141, a derivative of pBMBL, contained the *hmgA* gene (1,173 bp) amplified from the genomic DNA of BMB171 by using primers hmgA1 and hmgA2. The vector pBMB3144 carried the *hmgA* gene operon from BMB171, and the vector pBMB3145 carried the *hmgA* gene operon from BMB181. The *hmgA* gene operon region (about 3.9 kb) was amplified by using primers HMGAop-S and HMGAop-A. The vector pBMB3146, a derivative of pHT304, contained the transcription promoter region and the terminator region of the *hmgA* gene operon and the *hmgA* gene from BMB171. The promoter region (394 bp) was amplified from the genomic DNA of BMB171 by using primers HMGAop-S and HMGAop-A2. The *hmgA* gene (1,173 bp) and the terminator region (232 bp) of the *hmgA* gene operon was amplified from the genomic DNA of BMB171 by using primers HMGA-S2 and HMGAop-A.

Considering that the melanin production from homogentisate induced by a deficiency of HmgA in organisms is associated with the tyrosine metabolism pathway (**Figure 3A**), we analyzed the genome of BMB171 and found that BMB171 carried the genes encoding HppD, HmgA, and FahA (**Figure 3B**). The relationship between the biosynthesis of the pigment in the strain BMB181 and HmgA was tested by amplifying the *hmgA* gene from the BMB181 genomic DNA and sequencing. After aligning the inferred amino acid sequence of HmgA from the publicly available BMB171 sequences (He et al., 2010), a glycine was found to be replaced by a glutamate at residue 272 in HmgA in the pigmented strain BMB181.

Whether this amino acid substitution is responsible for the observed pigmented phenotype was tested by performing a complementation analysis. Specifically, the plasmid pBMB3141 containing the 171*hmgA* gene was transformed into BMB181 to create the transformant strain BMB3141 (171*hmgA^+^*), and BMB3142, a BMB181 derivative containing the plasmid pBMBL was used as a control. It was found that BMB3141 (171*hmgA^+^*) was reverted to a non-pigmented phenotype, with no significant increase observed in the OD_400_ of the supernatant relative to the control (**Figure 1B**). These results indicate that the G272E version of HmgA is responsible for melanin production in the *B. thuringiensis* pigmented strain BMB181.

### Disruption of HmgA Results in Pigment Formation Due to Inactivation of Homogentisate Dioxygenase

**FIGURE 4:**
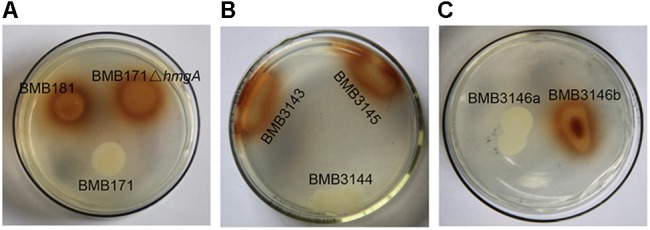
Phenotypes of *B. thuringiensis* strains grown on LB agar plates. **(A)** Phenotypes of BMB171, BMB181, and BMB171Δ*hmgA*. **(B)** Phenotypes of BMB3143, BMB3144, and BMB3145. BMB3143, a derivative of BMB171Δ*hmgA*, contained pHT304, as a negative control; BMB3144, a derivative of BMB171Δ*hmgA*, contained pBMB3144 harboring the *hmgA* gene operon from BMB171; and BMB3145, a derivative of BMB171Δ*hmgA*, contained pBMB3145 harboring the *hmgA* gene operon from BMB181. **(C)** Phenotypes of BMB3146a and BMB3146b. BMB3146a, a derivative of BMB181, contained pBMB3144; BMB3146b, a derivative of BMB181, contained pBMB3145.

To verify that the lack of a functional HmgA is responsible for the pigment formation of *B. thuringiensis*, a *hmgA* insertion mutant strain was constructed by homologous recombination. Specifically, the plasmid pBMB3143 containing *hmgA* gene fragments flanking the kanamycin resistance cassette was constructed and transferred into the strain BMB171. The recombinants with double-crossover homologous recombination integration in the resident *hmgA* gene were selected and verified by PCR. The strain carrying the *hmgA*::*kan* disruption, named BMB171Δ*hmgA*, was able to produce the brownish black pigment in cultures (**Figure 4A**). HPLC analysis showed that HGA was secreted and accumulated in the culture supernatants of BMB171Δ*hmgA* (**Figure 2D**).

The complete *hmgA* gene operons from the strains BMB171 and BMB181 were amplified and used for the complementation analysis, and the plasmids containing different alleles of *hmgA* were named as pBMB3144 (carrying the operon containing the *hmgA* gene from BMB171) and pBMB3145 (carrying the operon containing the *hmgA* gene from BMB181). The plasmids expressing either allele of *hmgA* were transferred into the strain BMB171Δ*hmgA* (pigmented phenotype). It was found that the transformant harboring the 171*hmgA* gene operon (strain BMB3144) exhibited a non-pigmented phenotype, while the transformant harboring the 181*hmgA* variant operon (strain BMB3145) remained pigmented (**Figure 4B**). The same result was found when the two plasmids were introduced into the strain BMB181 (**Figure 4C**). These data suggest that the HmgA variant from the BMB181 mutant with residue Gly272 replaced by the residue Glu is not functional and ultimately results in pigment overproduction, while the functional HmgA enzyme from the strain BMB171 results in the absence of pigment from HGA.

### Mutational Analysis of HmgA in *B. thuringiensis*

**FIGURE 5:**
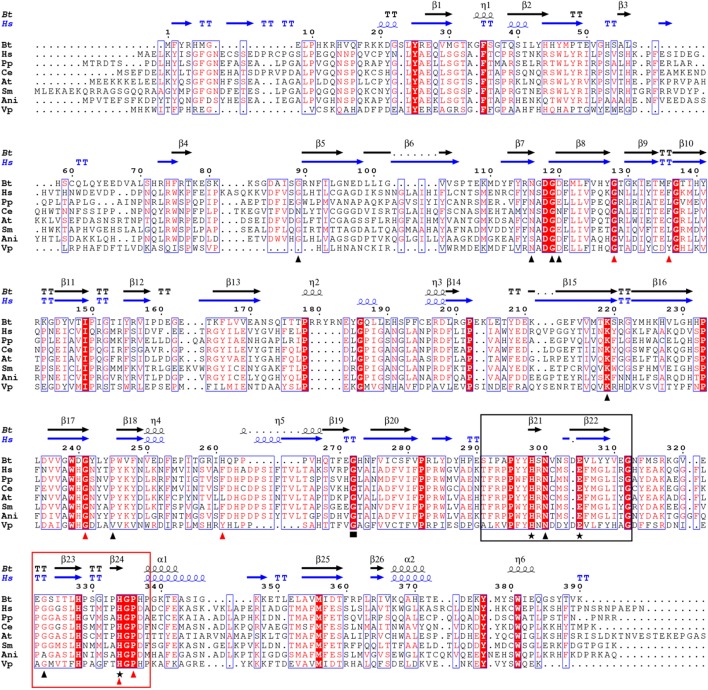
Sequence alignment of HmgA proteins from different organisms. The positions of the two conserved cupin motifs are boxed. Motif 1 and motif 2 are shown in the black box and red box, respectively. Amino acid substitutions in 171HmgA are marked by triangles. The red triangles point to the residues of 171HmgA that when replaced by alanine residues affect pigment production. The black triangles indicate that the residues of 171HmgA, when mutated to alanine had no effect on pigment production. The black stars indicate the conserved residues responsible for iron binding. The black rectangle shows the amino acid substitution (G272E) in BMB181. *B. thuringiensis*, homogentisate 1,2-dioxygenase from *B. thuringiensis* BMB171 (ADH05034.1); Hs, homogentisate 1,2-dioxygenase from *Homo sapiens* (CAA99340.1); Pp, homogentisate 1,2-dioxygenase from *Pseudomonas putida* (AAO12527.1); Ce, 2,5 dihydroxyphenylacetate oxidase from *Caenorhabditis elegans* (AAD00776.1); At, homogentisate 1,2-dioxygenase from *Arabidopsis thaliana* (AAD00360.1); Sm, homogentisate dioxygenase from *Sinorhizobium meliloti* (AAD29874.1); Ani, 2,5 dihydroxyphenylacetate oxidase from *Aspergillus nidulans* (AAC49071.1); and Vp, homogentisate 1,2-dioxygenase from *Vibrio parahaemolyticus* 10329 (EGF43769.1). The sequences were aligned with ClustalW (Chenna et al., 2003) and ESPript (Robert and Gouet, 2014).

To further test whether other single residue mutations have the same effect as G272E on melanin production, we designed 14 single-point mutations (G89A, N116A, G119A, D120A, G128A, F136A, K219A, G241A, P245A, H261A, N300A, G323A, H334A, and P336A) in 171HmgA based on the sequence alignment and the secondary structure of 171HmgA (**Figure 5**). A *trans*-complementation test was performed by introducing all the sequences under the control of the shuttle vector pHT304 separately into the pigmented strain BMB171Δ*hmgA*. Among the fourteen transformants, eight (containing G89A, N116A, G119A, D120A, K219A, P245A, N300A, and G323A, respectively) showed a non-pigmented phenotype, while the other six (containing G128A, F136A, G241A, H261A, H334A, and P336A, respectively) showed a pigmented phenotype (**Table 3**), implying that these six amino acid substitutions could result in the function loss of HmgA, making it unable to restore the non-pigment phenotype.

**Table 3:** Site-directed mutagenesis in HmgA.

Transformants of BMB171 (Δ*hmgA*)	Mutation position	Amino acid change	Phenotype^a^	Equivalent residue(s) in human HmgA	Amino acid changes in AKU patients (Zatkova, 2011; Usher et al., 2015)^b^
BMB3147	hmgA89	G89A	N	115G	G115R
BMB3148	hmgA116	N116A	N	149N	N149K
BMB3149	hmgA119	G119A	N	152G	G152A
BMB3150	hmgA120	D120A	N	153D	D153G
BMB3151	hmgA128	G128A	P	161G	G161R
BMB3152	hmgA136	F136A	P	169F	F169L
BMB3153	hmgA219	K219A	N	248K	K248E
BMB3154	hmgA241	G241A	P	270G	G270R
BMB3155	hmgA245	P245A	N	274P	P274L
BMB3156	hmgA261	H261A	P	290F	?
BMB3157	hmgA300	N300A	N	337N	N337D
BMB3158	hmgA323	G323A	N	360G	G360R/A
BMB3159	hmgA334	H334A	P	371H	H371R
BMB3160	hmgA336	P336A	P	373P	P373L

^a^*N: no pigment; P: pigment.*

^b^*?: no single amino acid substitution was found in the residue F290 in AKU patients in the present study*.

## Discussion

In aerobic organisms, L-Tyrosine degradation via homogentisate (HGA) is initiated by the conversion of tyrosine to 4-hydroxyphenylpyruvate by tyrosine aminotransferases, followed by the formation of HGA from 4-hydroxyphenylpyruvate by HppD and the oxidation of HGA to maleylacetoacetate (MAA) by HmgA. MAA is isomerized by maleylacetoacetate isomerase (MaiA) to fumarylacetoacetate, which is subsequently hydrolyzed by FahA to fumarate and acetoacetate (Schmaler-Ripcke et al., 2009; Valeru et al., 2009). The genes encoding these enzymes (HppD, HmgA, FahA, and MaiA) are adjacent on the chromosome of several organisms such as *Aspergillus fumigatus, Pseudomonas chlororaphis*, and *Vibrio cholerae* (Kang et al., 2008; Schmaler-Ripcke et al., 2009; Valeru et al., 2009). In the KEGG pathway database, three genes (BMB171_C1377, BMB171_C1351, and BMB171_C2647) are predicted to encode the aminotransferases involved in the conversion of L-tyrosine to 4-hydroxyphenylpyruvate. The three enzymes (HppD, HmgA, and FahA) in *B. thuringiensis* BMB171 are probably transcribed as part of an operon as indicated by the genomic analysis (**Figure 3B**). Analyzing the genome sequences of *B. thuringiensis* strains of different H serotypes, we found that the gene operon for L-Tyrosine degradation via HGA is located on every genome (data not shown). However, the genes responsible for the isomerization of MAA cannot be directly identified in *B. thuringiensis* by analyzing the genomic data. This implies that the genes responsible for the MAA metabolism of *B. thuringiensis* might be different from that of the reported genes. Even so, we found that the inactivation of HmgA leads to pyomelanin hyperproduction in *B. thuringiensis* strains. We speculate that *B. thuringiensis* strains have the potential to produce pyomelanin via HGA through the tyrosine metabolism pathway by inactivating HmgA.

HmgA is involved in the catabolism of the phenylalanine and tyrosine pathway. The structure of the human HmgA shows that the enzyme forms a hexamer arrangement consisting of a dimer of trimers (Titus et al., 2000). HmgA has been predicted to belong to the cupin-like superfamily^1^, which refers to a β-barrel structural domain, on the basis of the primary sequence. Two conserved histidine-containing motifs provide the signature sequence for the cupin superfamily (Dunwell et al., 2001). Despite the considerable variability of the HmgA proteins from different organisms in their primary amino acid sequences (from 22 to 65%), the residues involved in coordinating Fe^2+^ are highly conserved in the HmgA proteins from bacteria, fungi, plants, invertebrates, and humans (**Figure 5**). Three residues, including two residues His298 and Glu305 in motif 1, and one residue His334 in motif 2, are responsible for the metal ion binding of the 171HmgA protein (**Figure 5**). Additionally, the secondary structure of 171HmgA is composed of many β-strands (**Figure 5**). In this study, we found that a G272E mutation in HmgA resulted in pigment overproduction in the *B. thuringiensis* strain BMB181. Residues 272–274 of HmgA form a loop structure connecting two β-strands (β19 and β20). Gly272 is highly conserved in different HmgAs and the equivalent in the human HmgA is Gly309 (**Figure 5**). A G309V missense mutation has previously been found in AKU patients (Nemethova et al., 2016). These findings suggest that the residue Gly272 plays a very important role in the enzyme activity. To verify whether other residues in the loops or adjacent to the loops have the same effect as the residue Gly272, we designed 14 single-point mutants. Nine of the residues (Gly89, Gly119, Gly128, Phe136, Pro245, His261, Asn300, Gly323, and Pro336) are located in different loop regions, and the other five (Asn116, Asp120, Lys219, Gly241, and His334) are located at the start or end of different β-strands adjacent to the loop-forming residues. The result shows that only six mutants (G128A, F136A, G241A, H261A, H334A, and P336A) have an effect on melanin production (**Table 3**). The activity of the enzyme can be disrupted by mutations in many different ways. Some will directly or indirectly affect the active site, others will interfere with the folding of the subunit, and some will affect intersubunit interactions (Rodriguez et al., 2000). The relationship between these residues and the catalytic activity needs to be further confirmed by biochemical characterization and crystal structure.

Previous studies have shown that residues in many different sites of homogentisate 1,2 dioxygenase from humans are essential for enzyme function (Rodriguez et al., 2000; Vilboux et al., 2009; Zatkova, 2011). For instance, single amino acid substitutions at different positions of HmgA can result in pyomelanin overproduction in AKU patients (Rodriguez et al., 2000; Vilboux et al., 2009; Zatkova, 2011). The deduced amino acid sequence of 171HmgA consists of 390 amino acids and shares 24% identity with the HmgA from humans. The single-residue substitution in the 13 equivalents to the aforementioned 14 mutational residues in the human HmgA sequence would result in the loss of enzyme activity and pigment production (**Table 3**; Zatkova, 2011; Usher et al., 2015). These results indicate that five residues (G128, F136, G241, H334, and P336) are more conservative than the others (G89, N116, G119, D120, K219, P245, N300, and G323) in HmgA, and mutations in residues G128, F136, G241, H334, and P336 could affect the production of pyomelanin in both humans and *B. thuringiensis*.

Pyomelanin production has been studied in different bacteria. The accumulation of pyomelanin does not affect the growth characteristics nor the expression of key virulence factors of *B. anthracis* (Han et al., 2015). Notably, the mutant *P. aeruginosa* strain PA14Δ*hmgA* is significantly more virulent than the wild-type PA14 as PA14Δ*hmgA* can kill the nematodes at a greatly accelerated rate compared with the wild-type PA14 (Harmer et al., 2015). Additionally, pyomelanin has been identified as the primary melanin produced by the *A. media* strain WS through the autoxidation and self-polymerization of HGA (Wang et al., 2015). The melanin produced by the strain WS serves as an excellent photoprotective agent for BTI against UV and sunlight radiation (Wan et al., 2007). A previous study has shown that the pigment produced by the *B. thuringiensis* strain BMB181 protects against UV radiation (Liu et al., 2013). To test the possible role of pyomelanin in H_2_O_2_ resistance, we estimated the viability of the culture of the strain BMB171 under H_2_O_2_ treatment and found that the supernatant from the mutant culture was able to protect the vegetative cells from the effect of H_2_O_2_ based on the growth curves (data not shown). We speculate that the pyomelanin produced by *B. thuringiensis* strains may have a significant synergistic effect on the crystal proteins against nematodes and other insect pests by not affecting the growth and the expression of key virulence factors of *B. thuringiensis* and protecting *B. thuringiensis* cells from the stresses such as H_2_O_2_ and UV during the life cycle.

In this study, we constructed several plasmid vectors for complementation analysis (**Figure 3B**). The plasmid pBMB3141 was constructed using the vector pBMBL (unpublished data) and the single *hmgA* gene fragment for the complementation analysis. However, we found that the strain BMB181 could not completely restore the non-pigment phenotype with the plasmid pBMB3141 transformed into it, and the pigment appeared after culturing the transformant BMB3141 (a derivative of BMB181 containing pBMB3141) in LB medium for over 40 h (**Figure 1B**), suggesting that the promoter of the vector pBMBL, a sporulation-dependent promoter (BtI-BtII), is not suitable for this restoration test. To solve this problem, we tried to construct a plasmid vector that contained its own promoter and terminator region of the *hmgA* gene for complementation analysis. As shown in **Figure 3B**, the genes *hppD*, *fahA*, and *hmgA* are organized as an operon as indicated by the genomic analysis, and the transcription of the operon would be influenced with the *hmgA* gene being interrupted by the kanamycin resistance cassette in the strain BMB171Δ*hmgA*. Thus, we constructed the plasmid vectors (**Figure 3B**) that contained the complete *hmgA* gene operon from strains BMB171 and BMB181 separately for the complementation analysis (**Figures 4B,C**). The functions of HmgA variants in melanin production were also tested by the complementation analysis. We firstly constructed the vector pBMB3146 that contained the promoter and terminator regions of the *hmgA* gene operon and the *hmgA* gene (**Figure 3B**). All of the point mutations in HmgA were carried out by gene splicing by overlap extension PCR (SOE PCR) with pBMB3146 as the template. By introducing the plasmids containing different *hmgA* gene variants into the pigmented strain BMB171Δ*hmgA*, the roles of different HmgA variants was preliminarily determined by the phenotypes (pigmented or non-pigmented) (**Table 3**). This indicates that some of these residues play important roles in enzyme activity. Nevertheless, the exact roles of these residues for the enzyme HmgA could not be determined based only on the aforementioned data. The difference between HmgA variants should be distinguished by catalytic mechanisms in further related research.

In summary, *B. thuringiensis* strains could produce pyomelanin via HGA upon the deactivation of the HmgA or the disruption of the *hmgA* gene. We found that a G272E amino acid substitution in HmgA resulted in pigmentation. Several other residues in the loops or adjacent to the loops have the same effect as the residue Gly272 on the enzyme activity and melanin production. It is possible to generate more mutations in the *hmgA* gene and reintroduce them into *B. thuringiensis* and in many cases obtain a mutant phenotype (pigment overproducer). This approach has potential use to producing *B. thuringiensis* strains more resistant to UV. This is also an interesting model to study the human gene responsible for alkaptonuria.

## Author Contributions

WY, LR, and MS designed the research. WY and JT performed the research. WY, JZ, LR, DP, and MS analyzed the data. WY, LR, and MS wrote the manuscript.

## Conflict of Interest Statement

The authors declare that the research was conducted in the absence of any commercial or financial relationships that could be construed as a potential conflict of interest.

## Footnotes

1 https://www.ncbi.nlm.nih.gov/cdd/
